# 

**Published:** 1875-06

**Authors:** 


### Contents of June Number.

ART. I.—Ovariotomy by Enucleation, what it is and how to do it. By Julius F. Miner, M. D. .... 401
ART. II.—Tracheotomy in Croup. By W. C. Phelps, M. D. .... 405
ART. III.—Syphilitic Hydrosarcocele. By B. J. Preston, M. D. .... 408
Correspondence, .... 410

MISCELLANEOUS.

Ohio State Medical Society, .... 415
Relation of the Profession to the Secular Press and the Rostrum .... 420
A Case of Myelitis Ending in Recovery, .... 429
A Case of Worms in the Urinary Bladder, .... 431
The Sense of Taste, .... 431
Transfusion, .... 433
On Fibroids of the Uterus, .... 433

EDITORIAL DEPARTMENT.

The Medical Profession in California, .... 434
Items, .... 435

BOOKS REVIEWED:

The Histology and Histo-Chemistry of Man. By Heinrich Frey, .... 437
Orthopædia. By James Knight, M. D. .... 437
Syphilitic Lesions of the Osseous System in Infants. By R. W. Taylor, M. D. .... 438
Lectures on Diseases of the Respiratory Organs, Heart and Kidneys. By Alfred L. Loomis, M. D. .... 439

Books and Pamphlets Received, .... 440

